# LncRNA small nucleolar RNA host gene 1 (SNHG1) mediates acidic bile salt-induced EMT via the ULK1-Notch1 axis in Barrett’s esophagus

**DOI:** 10.1186/s43556-025-00285-4

**Published:** 2025-07-09

**Authors:** Jianfeng Zhou, Rongyan Zhao, Zixiang Li, Xuelan Ma, Wenke Jin, Yong Yuan, Ning Li, Bo Liu, Yushang Yang

**Affiliations:** 1 Department of Thoracic Surgery, Department of Biotherapy, Cancer Center and State Key Laboratory of Biotherapy, West China Hospital, Sichuan University, Chengdu, 610041 China; 2 Sichuan Engineering Research Center for Biomimetic Synthesis of Natural Drugs, School of Life Science and Engineering, Southwest Jiaotong University, Chengdu, 610031 China; 3 School of Traditional Chinese Materia Medica, Key Laboratory of Innovative Traditional Chinese Medicine for Major Chronic Diseases of Liaoning Province, Key Laboratory for TCM Material Basis Study and Innovative Drug Development of Shenyang City, Shenyang Pharmaceutical University, Shenyang, 110016 China

**Keywords:** Barrett’s esophagus, Small nucleolar RNA host gene 1 (SNHG1), UNC-52-like kinase 1 (ULK1), Notch1, Epithelial-mesenchymal transition (EMT)

## Abstract

Barrett’s esophagus (BE) is a precancerous condition closely linked to chronic gastroesophageal reflux disease, characterized by the abnormal transformation of esophageal squamous mucosa into specialized intestinal-type epithelium, significantly elevating the risk of esophageal adenocarcinoma (EAC). Recurrent acidic bile reflux promotes epithelial-mesenchymal transition (EMT), a critical event driving malignant progression. However, the underlying molecular mechanisms remain incompletely understood. Here, we identify the long non-coding RNA small nucleolar RNA host gene 1 (SNHG1) as a novel regulator of EMT in BE, mediating its effects through the UNC-52-like kinase 1 (ULK1)-Notch1 signaling axis and autophagy modulation. Using BAR-T and CP-C cell models, we demonstrate that SNHG1 expression is elevated following acidic bile salt exposure, enhancing EMT characteristics by promoting the phosphorylation of ULK1 and activating Notch1 signaling. Pharmacological interventions targeting autophagy (Rapamycin) and Notch signaling (DAPT) further confirmed that SNHG1’s effects on EMT are mediated via modulation of the autophagy-Notch1 interplay. We further validated our results in vivo using the previously described IL1β-induced Lgr5-CreERT2; p16^flox/flox^/Kras^G12D^ mouse model, which reliably reproduces the histological progression of Barrett’s-like dysplasia in the squamocolumnar junction (SCJ), confirming SNHG1’s critical role in regulating EMT and BE progression. Additionally, SNHG1 expression was significantly elevated in patients who progressed to low- or high-grade dysplasia, as confirmed by diagnostic endoscopic biopsies. Collectively, our study uncovers SNHG1 as a central molecular mediator linking acidic bile-induced EMT and autophagy regulation via the ULK1-Notch1 axis, highlighting its potential as a therapeutic target for preventing BE recurrence and progression to EAC.

**Supplementary Information:**

The online version contains supplementary material available at 10.1186/s43556-025-00285-4.

## Introduction

Barrett’s esophagus (BE) is a premalignant condition in which the normal stratified squamous epithelium lining the distal esophagus is replaced by specialized intestinal-type columnar epithelium, primarily as a result of chronic exposure to gastric acid and bile reflux from long-standing gastroesophageal reflux disease (GERD). BE is estimated to affect up to 1.6% of the general population globally [1], with a higher prevalence in Western countries—particularly among middle-aged to older white males with chronic GERD symptoms [2]. In the United States, for example, the prevalence of BE among individuals undergoing endoscopy for reflux symptoms may reach as high as 10–15%. While the annual incidence of progression from non-dysplastic BE to esophageal adenocarcinoma (EAC) is relatively low—approximately 0.1% to 0.5% per year—the lifetime risk remains substantial, especially in patients with additional risk factors such as male sex, smoking history, central obesity, and confirmed dysplasia [3, 4]. EAC is a highly aggressive cancer with poor prognosis, and BE remains the only well-established precursor lesion for its development. Accordingly, clinical guidelines recommend that patients with confirmed BE undergo regular endoscopic surveillance to monitor dysplasia and early neoplastic transformation. Once dysplasia is detected, endoscopic eradication therapies—most notably radiofrequency ablation (RFA)—are considered the gold standard for preventing progression to invasive cancer due to their minimally invasive nature and favorable safety profile [5]. However, even after successful initial treatment, recurrent intestinal metaplasia is observed in approximately 10% of patients per year [6].

The precise mechanisms underlying the recurrence of Barrett’s metaplasia after RFA remain elusive; however, sub-squamous intestinal metaplasia (SSIM) has been proposed as a potential cause [7, 8]. SSIM refers to intestinal metaplastic glands situated beneath the squamous epithelium within the esophageal lamina propria, often evading detection by conventional endoscopic methods [9]. RFA treatment is designed to induce controlled thermal injury, effectively disrupting the superficial metaplastic epithelium while preserving the submucosa to minimize the risk of esophageal strictures [10]. Consequently, this technique may inadequately target SSIM glands that lie deeper beneath the protective squamous epithelial layer, potentially contributing to disease recurrence [11]. Nevertheless, the pathogenesis and molecular regulation of SSIM remain poorly understood.

A critical pathogenic mechanism proposed for SSIM involves the migration of Barrett’s epithelial cells into the squamous lamina propria [12]. In this context, Barrett’s epithelial cells acquire mesenchymal properties through epithelial-mesenchymal transition (EMT), enabling penetration into the underlying tissue before potentially reverting to an epithelial state, thereby initiating SSIM formation. EMT in Barrett’s cells is frequently induced by chronic exposure to acidic bile salts, as observed in both cell-culture experiments and animal models of reflux esophagitis induced by esophagojejunostomy [11, 13]. These conditions stimulate EMT via activation of several key pathways, notably the vascular endothelial growth factor (VEGF) signaling pathway, which involves the zinc finger E-box-binding homeobox proteins (ZEB1/2) and transcription factors such as Snail and Slug, which suppress epithelial markers like E-cadherin (CDH1) [13–15]. Despite these observations, the specific molecular events underlying acidic bile salt-induced EMT and its implications for SSIM development require further elucidation. The recurrence of BE following treatment presents not only a major challenge in clinical management but also underscores the urgent need to deepen our understanding of the cellular and molecular mechanisms underlying BE persistence and relapse. In particular, the roles of EMT and SSIM as potential"hidden reservoirs"warrant focused investigation. Elucidating these processes may offer novel therapeutic targets and strategies for precision treatment and long-term management of BE patients.

In the present study, we investigated the effects of prolonged exposure to acidic bile salts on EMT in Barrett’s epithelial cells, with particular attention to the underlying molecular mechanisms. Our findings highlight the Notch signaling pathway as a crucial mediator of EMT regulation in Barrett’s epithelial cells, notably influencing autophagy levels through modulation of UNC-52-like kinase 1 (ULK1) phosphorylation. Furthermore, we identified the long non-coding RNA (lncRNA) small nucleolar RNA host gene 1 (SNHG1) as a central regulatory molecule involved in modulating the ULK1-Notch1 signaling axis, thereby orchestrating the EMT process. These results provide significant new insights into the molecular mechanisms driving Barrett’s esophagus pathogenesis and progression toward malignancy.

## Results

### Acidic bile salts induce epithelial-mesenchymal transition in Barrett’s esophagus through the Notch1 signaling pathway

EMT is recognized as a critical process in the progression from BE to EAC, yet the precise upstream mechanisms driving EMT in BE remain poorly defined [16]. While previous studies have demonstrated that acidic bile salts (ABS) can induce EMT-like features in BE, primarily through inflammatory and other signaling pathways, the specific molecular regulators underlying this process are not well-characterized [13, 17]. Notably, although Notch1 signaling has emerged as a prominent regulator of EMT in various cancers and fibrotic diseases, its role in mediating EMT within Barrett’s esophagus has not been clearly established, representing a significant gap in current research.

**Fig. 1:**
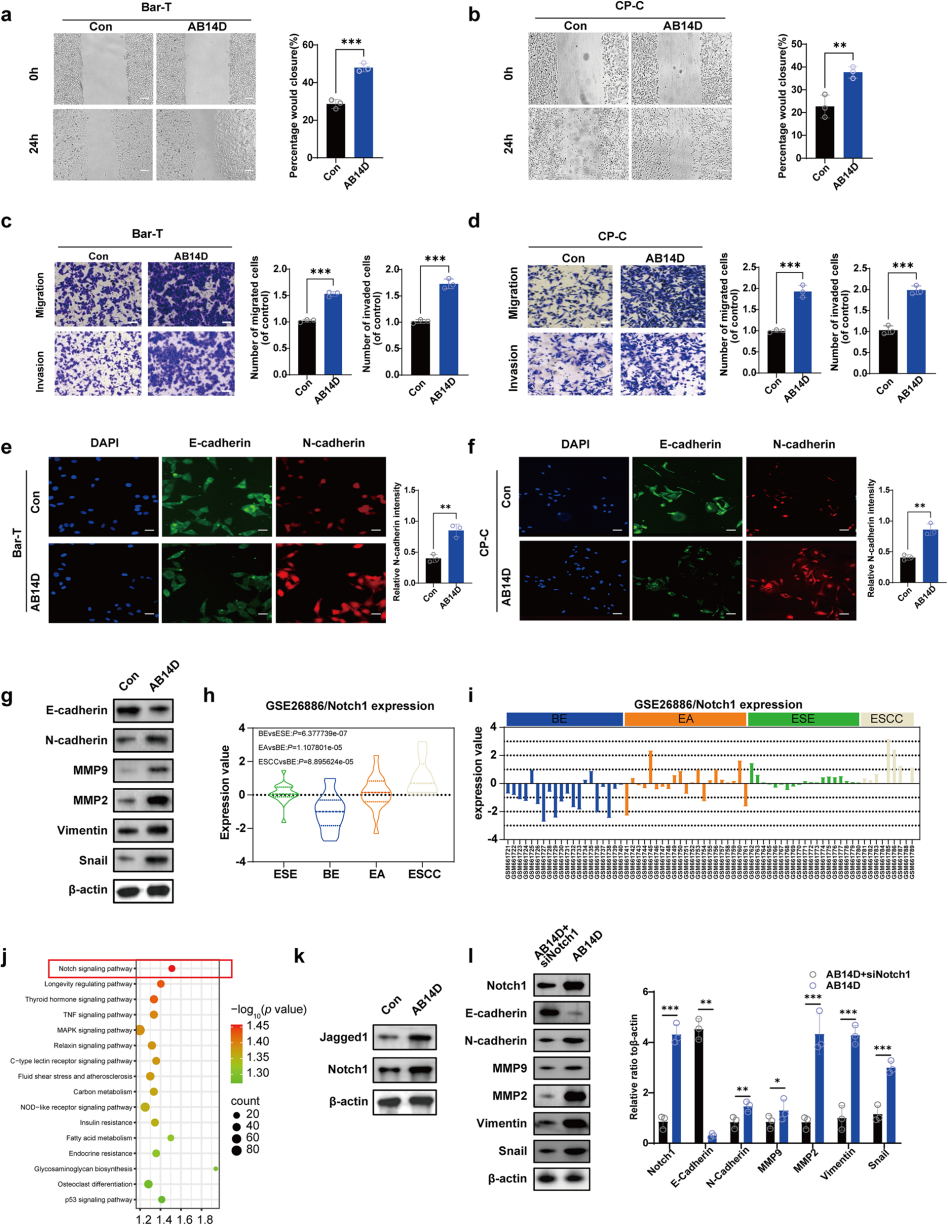
The regulation of EMT features in Barrett’s epithelial cells exposed to acidic bile salt is attributed to Notch1. **a**,**b** The scratch method was used to evaluate the capacity of cells to migrate. Scale Bar, 200 µm. **c**,**d** The number of migratory and invasive cells was measured by trans-well analysis. Scale Bar, 100 µm. **e**,**f** N**-**cadherin’s (shown in red) fluorescence intensity was evaluated by immunofluorescence analysis, which was then quantitatively analyzed. DAPI labeling was utilized to make the cell nuclei visible (blue). Scale Bar, 10 µm. **g** Using β-actin as a reference, Western blot analysis was used to assess the protein expression levels of N-cadherin, Vimentin, MMP2, snail, and MMP9. **h**, **i** Publicly available data (GSE26886) demonstrate that Notch1 expression is downregulated in BE compared to normal ESE but reactivated in esophageal adenocarcinoma (EAC) tissues. **j** KEGG enrichment analysis of Publicly available data (GSE26886) between BE and EAC. **k** Using β-actin as a control, Western blot analysis evaluated the protein expression of Jagged1 and Notch1. **l** Silencing Notch1 reverses ABS-induced EMT, confirming the critical role of Notch1 in driving EMT and invasiveness in Barrett’s esophagus cells. The control (Con) conditions refer to the absence of AB14D treatment. We used one-way ANOVA with Tukey’s post hoc test for multiple group comparisons, and Student’s t-test or Mann-Whitney U test for two-group comparisons. The values are the means ± standard error of at least three separate studies

To address this gap, we first verified the induction of EMT by treating Barrett’s epithelial cells with acidic bile salt medium for 30 minutes a day for 14 days (AB14D). Subsequent assays confirmed significant increases in migration and invasion capabilities (Fig. 1a-d), coupled with hallmark EMT marker changes: reduced E-cadherin and enhanced expression of N-cadherin, Vimentin, MMP-2, MMP-9, and nuclear Snail (Fig. 1e-g). These findings reinforced that acidic bile salts robustly promote EMT phenotypes in Barrett’s epithelial cells. We then explored Notch1 signaling, considering its known regulatory function in EMT across other contexts. Analysis of publicly available data (GSE26886) revealed Notch1 is significantly downregulated in BE compared to normal esophageal squamous epithelium (ESE) but becomes reactivated in EAC tissues (Fig. 1h, i), highlighting its potential dual role as a biomarker and a regulator during disease progression. To further substantiate the rationale for selecting the Notch signaling axis as our research focus, we conducted pathway enrichment analysis based on differentially expressed genes (DEGs) from the GSE26886 dataset, comparing BE tissues with EAC. As shown in Fig. 1j, the Notch signaling pathway ranked highest among all significantly enriched KEGG pathways, exhibiting the greatest fold enrichment and statistical significance (adjusted *P* < 0.05). While other EMT-related pathways such as TNF, MAPK, and thyroid hormone signaling also showed modest enrichment, their association was less robust compared to Notch signaling. This finding highlights the Notch pathway as a dominant upstream regulatory mechanism in BE progression. Our experiments demonstrated significant activation of Jagged1/Notch1 signaling upon acidic bile salt treatment (Fig. 1k). Crucially, silencing Notch1 effectively reversed ABS-induced EMT, confirming Notch1’s essential regulatory role in driving EMT phenotypes and invasiveness in Barrett’s esophagus cells (Fig. 1l).

Collectively, these results establish the novel involvement of Notch1 signaling in mediating EMT induced by acidic bile salts in BE, addressing an important gap in understanding Barrett’s esophagus progression and providing potential new avenues for therapeutic intervention.

### Autophagy modulates acidic bile salt-induced EMT via Notch1 signaling in Barrett’s epithelial cells

Autophagy has previously been implicated in regulating EMT processes via modulation of the Notch1 signaling pathway [18]. Given this potential regulatory role, we hypothesized a connection between autophagy suppression and Notch1 signaling during ABS-induced EMT in Barrett’s epithelial cells.

**Fig. 2:**
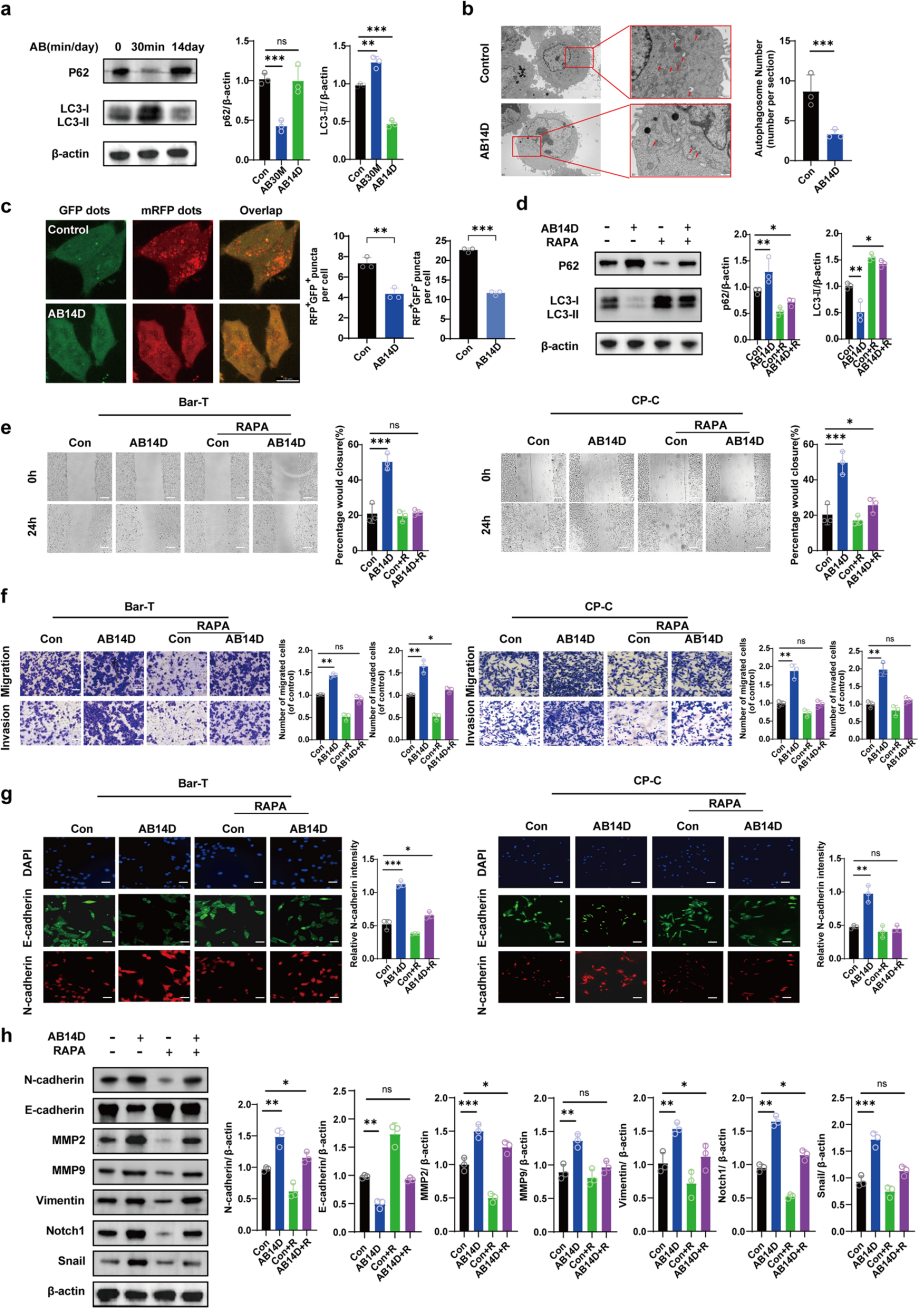
Autophagy inhibits EMT in acidic bile salt treatment Barrett’s epithelial cells by Notch1 signaling. **a** Western blot analysis using β-actin as a control was used to evaluate the expression of the p62 and LC3 proteins. **b**,**c** Electron microscopy and LC3 autophagic flux assays further confirm reduced autophagic vesicle formation in AB14D-treated cells. **d** Using β-actin as a reference, Western blot analysis revealed the expression of p62 and LC3 proteins after RAPA therapy. **e** Following RAPA treatment, the scratch technique was used to assess the cell migratory ability. Scale Bar, 200 µm. **f** After receiving RAPA therapy, the cells’ ability to migrate and invade was ascertained using transwell examination. Scale Bar, 100 µm. **g** After RAPA therapy, immunofluorescence analysis evaluated the fluorescence intensity of N-cadherin (shown in red) and performed a quantitative analysis on it. The blue-colored DAPI labeling made the cell nuclei visible. Scale Bar, 10 µm. **h** Using β-actin as a reference, Western blot analysis assessed the protein expression of Vimentin, MMP-2, MMP-9, N-cadherin, Notch1, Snail, and E-cadherin after RAPA therapy. The control (Con) conditions refer to the absence of AB14D treatment. We used one-way ANOVA with Tukey’s post hoc test for multiple group comparisons, and Student’s t-test or Mann-Whitney U test for two-group comparisons. Values represent mean values ± SEM from at least three independent experiments

To test this hypothesis, we evaluated autophagy activity in Barrett’s epithelial cells exposed to acidic bile salts for either a short (30 minutes, AB30M) or prolonged period (AB14D). Western blot analysis revealed that short-term exposure (AB30M) significantly reduced p62 levels and modestly increased LC3-II expression compared to control cells, suggesting enhanced autophagic flux under acute acidic bile stimulation. However, under chronic exposure (AB14D), LC3-II levels were significantly reduced, while p62 levels returned to near-baseline, indicating suppressed autophagy under chronic ABS exposure (Fig. 2a). Electron microscopy and LC3 autophagic flux assays further confirmed reduced autophagic vesicle formation in AB14D-treated BAR-T cells (Fig. 2b, c). Subsequently, we investigated whether reversing this suppression by activating autophagy could mitigate the EMT phenotype. Western blot analyses demonstrated that Rapamycin (RAPA), an autophagy activator, effectively restored autophagy, significantly elevating LC3-II levels and reducing SQSTM1/p62 expression compared to untreated controls (Fig. 2d). Functionally, RAPA-treated BAR-T and CP-C cells exhibited significantly lower migration rates in scratch wound-healing assays (Fig. 2e) and decreased invasion capacities in transwell assays (Fig. 2f) compared to cells exposed solely to acidic bile salts. Immunofluorescence assays showed notably reduced N-cadherin expression after RAPA treatment (Fig. 2g). Further Western blot analysis confirmed that, under the conditions of EMT induced by AB14D, RAPA activated autophagy, upregulating the epithelial marker E-cadherin and significantly downregulating the expression of mesenchymal markers N-cadherin, MMP2, MMP9, and Vimentin (Fig. 2h). Additionally, under the conditions of EMT induced by AB14D, RAPA-mediated autophagy activation significantly inhibited Notch signaling, as evidenced by reduced levels of Notch1 and Snail proteins (Fig. 2h).

Collectively, these findings indicate that suppression of autophagy is necessary for ABS-induced EMT, and reactivation of autophagy effectively inhibits this process by modulating the Notch1 signaling pathway. Thus, targeting autophagy presents a promising therapeutic strategy for preventing EMT and its associated progression in Barrett’s esophagus.

### ULK1-Notch1 axis participates in Barrett’s epithelial cell EMT stimulated by acidic bile salts

**Fig 3:**
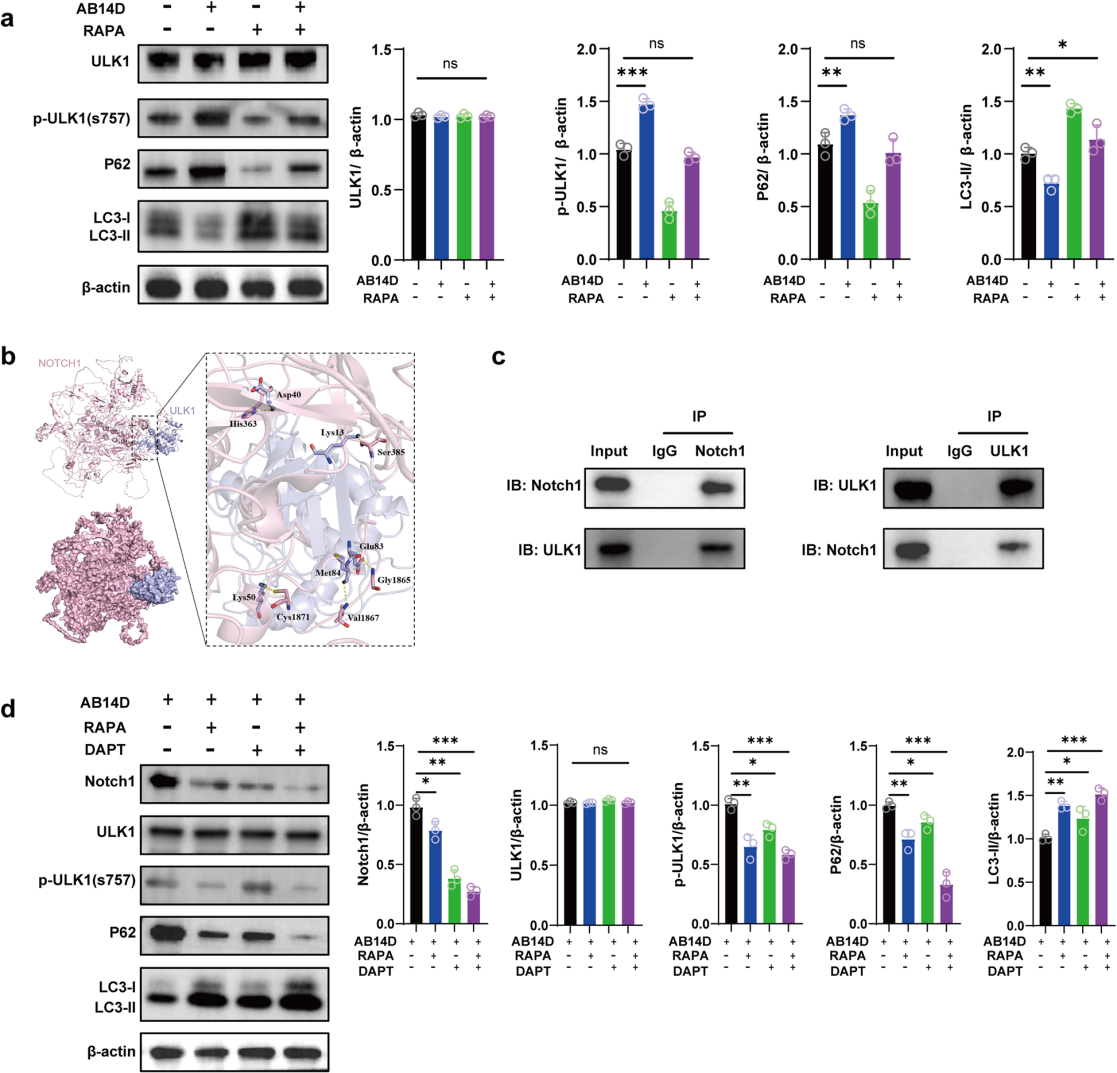
DAPT controls autophagy by means of the ULK1-Notch1 pathway. **a** Western blot analysis using β-actin as a control was used to evaluate the protein expression of ULK1, p-ULK1(S757), p62, and LC3 after RAPA therapy. **b** Protein-protein docking analyses reveal a strong interaction between ULK1 and Notch1, with a docking score of 99.059. Visualizations show hydrogen bonds between specific residues of Notch1 and ULK1, indicating a potential physical interaction. **c** Following stimulation with acidic bile salt, Notch1 and ULK1 in Barrett’s epithelial cells were shown to physically interact, as demonstrated by Co-IP tests. **d** Using β-actin as a reference, Western blot analysis assessed the protein expression of ULK1, p-ULK1(S757), p62, and LC3 after treatment with RAPA or the γ-secretase inhibitor DAPT. We used one-way ANOVA with Tukey’s post hoc test for multiple group comparisons, and Student’s t-test or Mann-Whitney U test for two-group comparisons. The values are the means ± standard error of at least three separate studies

ULK1 plays a crucial role in autophagy initiation, serving as a primary target of mTOR-mediated phosphorylation. RAPA, a potent autophagy activator, inhibits mTOR and consequently regulates ULK1 phosphorylation, thereby facilitating autophagy induction [19]. In our experiments, Western blot analyses revealed a significant decrease in p-ULK1 (Ser757) expression following RAPA treatment compared to untreated control cells (Fig. 3a), confirming effective autophagy activation.

To further investigate the potential link between autophagy and Notch1 signaling through ULK1, we conducted protein-protein docking analyses using Discovery Studio 4.0 software with the ZDOCK module. The calculated docking score between NOTCH1 and ULK1 proteins was 99.059, indicative of a strong interaction. Docking visualizations identified hydrogen bonds formed between NOTCH1 residues (His363, Ser385, Gly2865, Val1867, and Cys1871) and ULK1 residues (Asp40, Lys13, Glu83, Met84, and Lys50) (Fig. 3b). Co-immunoprecipitation (Co-IP) assays further validated the physical interaction between Notch1 and ULK1 proteins in Barrett’s epithelial cells (Fig. 3c). To assess the functional role of Notch1 signaling in regulating ULK1 phosphorylation and autophagy in Barrett’s epithelial cells, we used DAPT, a γ-secretase inhibitor known to effectively suppress Notch activation by blocking its cleavage [20]. Western blot analysis demonstrated that treatment with either RAPA or DAPT alone significantly elevated LC3 expression while reducing Notch1, p-ULK1, and p62 levels in acidic bile salt (AB14D)-treated Barrett’s epithelial cells (Fig. 3d). Notably, the combined administration of RAPA and DAPT amplified these effects, indicating even more pronounced reductions in Notch1, p-ULK1, and increased LC3 compared to single treatments (Fig. 3d). These results suggest that DAPT-mediated Notch1 inhibition blocks acidic bile salt-induced ULK1 phosphorylation, thus promoting autophagy activity.

In summary, our findings demonstrate that the ULK1-Notch1 axis plays a central role in mediating acidic bile salt-induced EMT in Barrett’s epithelial cells by modulating autophagy. Specifically, ULK1 directly interacts with Notch1, and their phosphorylation interplay governs autophagy suppression and EMT promotion. Pharmacological inhibition of Notch signaling (DAPT) or mTOR signaling (RAPA) not only disrupts ULK1 phosphorylation but also enhances autophagic activity and mitigates EMT features. These results highlight the ULK1-Notch1 signaling axis as a critical regulatory hub in the pathogenesis of Barrett’s esophagus.

### SNHG1 clinically correlates with Barrett’s progression and mediates the interaction between ULK1 and Notch1

**Fig. 4:**
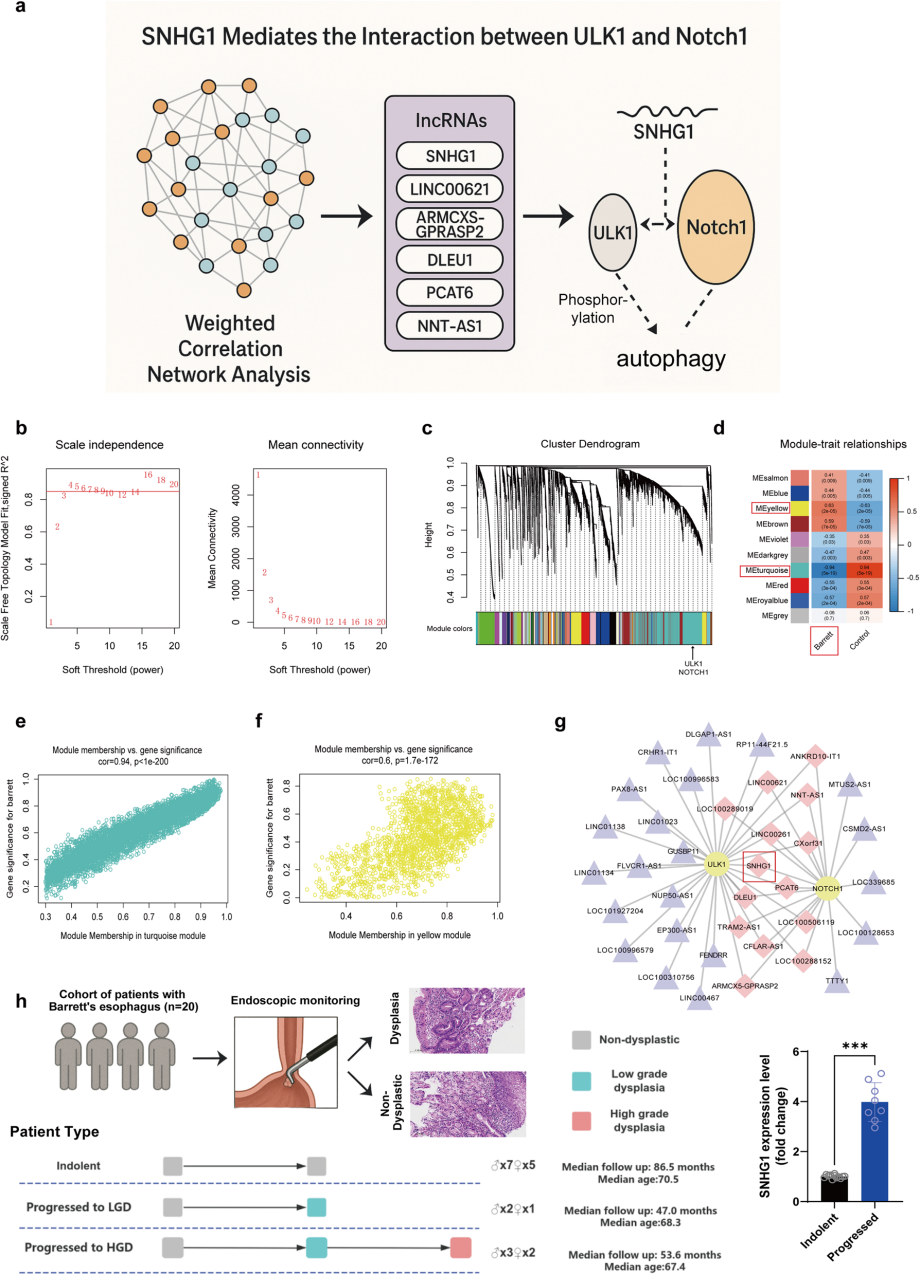
Construction of the core regulatory network for Barrett’s esophagus patients. **a** Workflow illustrating the identification of lncRNAs associated with BE progression through WGCNA and RIP-seq integration. **b** Scale independence and mean connectivity under each power. **c** The cluster dendrogram of each module. **d** The relationships of module-trait (correlation greater than 0.3). **e**, **f** Correlation between yellow and turquoise module membership and gene significance for Barrett. **g** The lncRNA subnetwork that regulated by ULK1 and Notch1. **h** Clinical validation of SNHG1 expression in BE patient biopsies (*n* = 20). Patients were stratified based on follow-up histological progression into indolent (non-progressors), LGD, and HGD groups. SNHG1 was significantly upregulated in patients progressing to LGD or HGD (*P* < 0.05), as assessed by qPCR

LncRNAs function as regulators of gene expression, exerting diverse effects on numerous crucial physiological processes. These effects include acting as chromatin modification factors, X chromosome inactivation factors, enhancers, transcriptional regulatory factors, and post-transcriptional regulatory factors, among others [21, 22]. Certain lncRNAs are known to interact with ULK1, potentially influencing its modification and function [23]. Therefore, we performed weighted correlation network analysis (WGCNA) to identify critical eigengenes associated with the initiation and progression of BE and to construct a regulatory network (Fig. 4a).

For network construction, we selected a soft threshold of 4 to ensure a scale-free distribution (Fig. 4b). The WGCNA analysis yielded 49 distinct gene modules (Fig. 4c). ULK1 and Notch1 were found within the turquoise module, which showed a strong correlation with BE traits (correlation = −0.94, *P* = 5×10^−19; Fig. 4c, d). To explore lncRNAs regulated by ULK1 and Notch1 and their potential roles in BE progression, we constructed a regulatory network using genes from the turquoise and yellow modules, both demonstrating high module membership and gene significance for BE (Fig. 4e, f). From this regulatory network, we extracted the lncRNA subnetwork regulated by both ULK1 and Notch1, identifying 14 lncRNAs highly correlated with BE progression (Fig. 4g). We further intersected these 14 candidate lncRNAs with 822 lncRNAs identified from RNA immunoprecipitation (RIP) sequencing targeting ULK1. This intersection identified six candidate lncRNAs: SNHG1, LINC00621, ARMCX5-GPRASP2, DLEU1, PCAT6, and NNT-AS1. However, qPCR validation in acidic bile salt-treated Barrett’s epithelial cells revealed that only SNHG1 exhibited significantly altered expression compared to control cells. These findings led us to hypothesize that SNHG1 mediates the interaction between ULK1 and Notch1, potentially influencing BE progression (Supplementary Fig. 1). To further substantiate the clinical relevance of SNHG1 in Barrett’s esophagus progression, we analyzed SNHG1 expression in a well-characterized cohort of BE patients (*n* = 20) who underwent longitudinal endoscopic surveillance (Fig. 4h). Based on histological evaluation and clinical follow-up, patients were classified into three categories: indolent (non-progressors), progressed to low-grade dysplasia (LGD), and progressed to high-grade dysplasia (HGD). Importantly, quantitative PCR analysis of endoscopic biopsy samples revealed that SNHG1 expression was significantly upregulated in patients who progressed to LGD or HGD compared to the indolent group. These findings further validate the association between elevated SNHG1 expression and BE progression, reinforcing its potential utility as a biomarker for risk stratification in clinical settings.

In summary, this section identifies SNHG1 as a clinically and mechanistically relevant lncRNA that modulates the interaction between ULK1 and Notch1, and is significantly upregulated in BE patients who later developed dysplasia. These findings support SNHG1 as a critical molecular of disease progression in Barrett’s esophagus.

### SNHG1 regulates ULK1-Notch1 axis-mediated autophagy inhibition and EMT activation in Barrett’s epithelial Cells

**Fig. 5:**
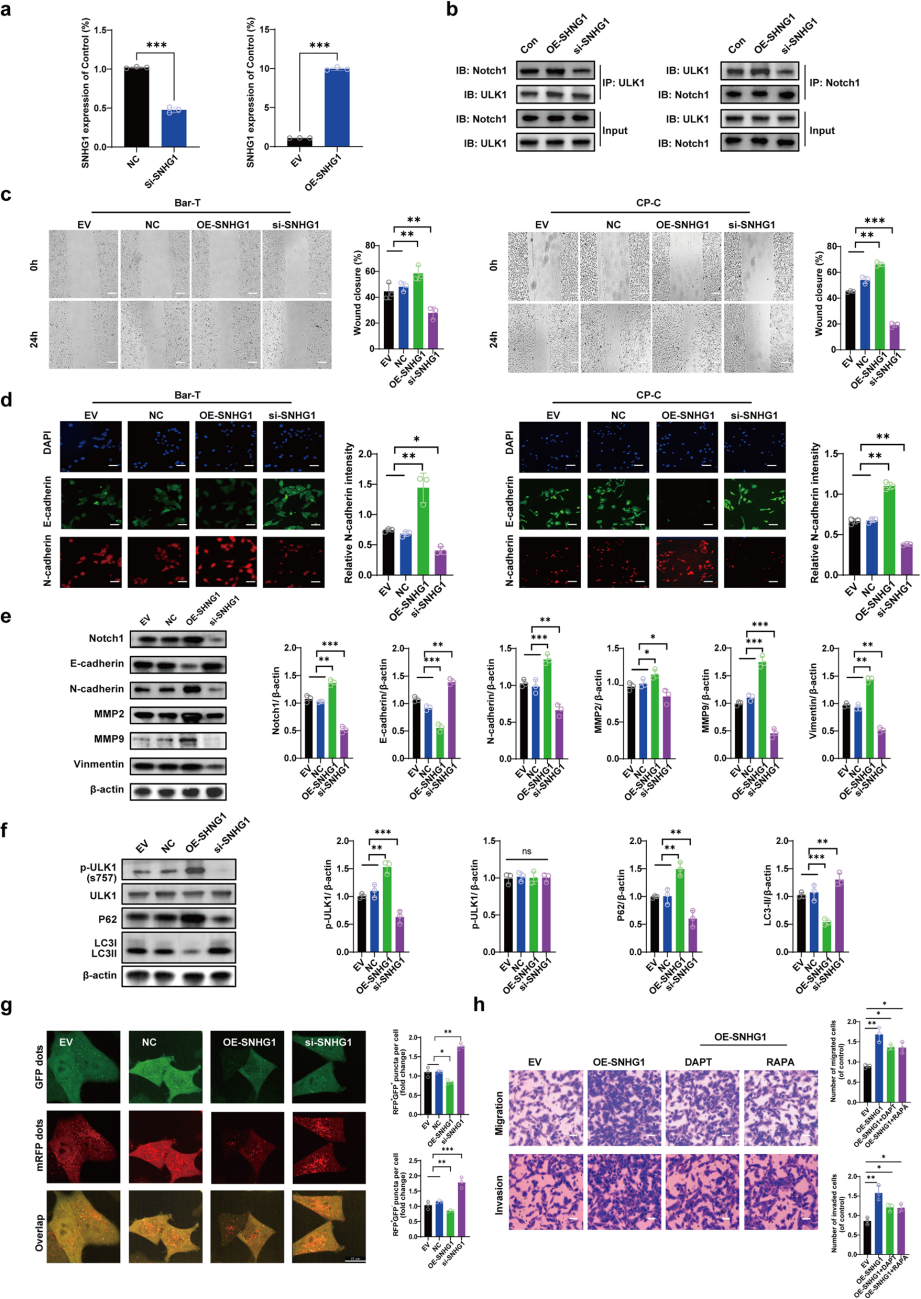
SNHG1 inhibits autophagy and activates EMT through modulation of the ULK1-Notch1 axis in Barrett’s epithelial cells. **a** The overexpression and silence efficiency of SNHG1. **b** Following SNHG1 overexpression or knockdown, Co-IP tests verified the physical connection between Notch1 and ULK1 in Barrett’s epithelial cells. **c** The scratch experiment evaluated the ability of cells to migrate when SNHG1 was overexpressed or knocked down. Scale Bar, 200 µm. **d** After overexpressing or knocking down SNHG1, immunofluorescence analysis detected the fluorescence intensity of N-cadherin (shown in red) and quantitatively analyzed it. The blue-colored DAPI labeling made the cell nuclei visible. Scale Bar, 10 µm. **e** Western blot analysis used β-actin as a reference to assess the protein expression of Notch1, E-cadherin, N-cadherin, MMP2, MMP9, and Vimentin after SNHG1 was overexpressed or knocked down. **f** Western blot analysis used β-actin as a reference to assess the protein expression of p-ULK1, ULK1, p62, and LC3 after SNHG1 was overexpressed or knocked down. **g** LC3 autophagic flux assays further support these findings. These results suggest that SNHG1 inhibits autophagy in BAR-T cells. **h** The migratory and invasive potential of cells after SNHG1 overexpression can be reversed by the DAPT or RAPA when assessed by transwell analysis. Scale Bar, 100 µm. The empty vector (EV) serves as the overexpression control. Normal control (NC) refers to normal control, which is the siNotch1 control. We used one-way ANOVA with Tukey’s post hoc test for multiple group comparisons, and Student’s t-test or Mann-Whitney U test for two-group comparisons. The values are the means ± standard error of at least three separate studies

We observed increased transcription levels of SNHG1 in Barrett’s epithelial cells upon exposure to acidic bile salts (Supplementary Fig. 1). Weighted correlation network analysis (WGCNA) suggested that SNHG1 participates in the ULK1-Notch1 signaling axis. To validate the physical association between SNHG1 and the ULK1-Notch1 axis, we performed Co-IP assays, which demonstrated that SNHG1 modulates the interaction between Notch1 and ULK1 (Fig. 5a, b). Specifically, SNHG1 overexpression enhanced the interaction, while its knockdown weakened the binding affinity between ULK1 and Notch1.

Based on these findings, we hypothesized that SNHG1 might regulate EMT characteristics in Barrett’s epithelial cells. To test this hypothesis, we evaluated cell migration using scratch wound-healing assays. Enhanced SNHG1 expression significantly increased cell migration compared to controls, whereas SNHG1 knockdown via siRNA markedly inhibited cell migration (Fig. 5c). These results indicate that SNHG1 promotes EMT in Barrett’s epithelial cells. Immunofluorescence assays further supported this conclusion, showing substantially decreased N-cadherin expression after SNHG1 knockdown and elevated N-cadherin levels following SNHG1 overexpression (Fig. 5d).

We next assessed EMT-related protein expression by Western blot. Overexpression of SNHG1 led to decreased E-cadherin and increased levels of Notch1, N-cadherin, MMP2, MMP9, and Vimentin compared to control groups (Fig. 5e). Conversely, SNHG1 silencing significantly increased E-cadherin expression and decreased Notch1, N-cadherin, MMP2, MMP9 and Vimentin (Fig. 5e). Moreover, Western blot analyses revealed that SNHG1 overexpression significantly increased p-ULK1 and p62 while decreasing LC3 levels. The opposite pattern was observed upon SNHG1 knockdown (Fig. 5f). LC3 autophagic flux experiments provided further validation of these findings (Fig. 5g). Importantly, re-expression of SNHG1 in siSNHG1-transfected cells restored EMT and autophagy phenotypes, confirming the specificity of the knockdown and excluding off-target effects (Supplementary Fig. 2). Collectively, these results demonstrate that SNHG1 inhibits autophagy and promotes EMT by modulating the ULK1-Notch1 axis in Barrett’s epithelial cells. To further validate the regulatory relationship between SNHG1 and ULK1-Notch1 expression, we conducted rescue experiments. We found that the EMT enhancement induced by SNHG1 overexpression was reversed upon RAPA-mediated autophagy activation or DAPT-induced Notch1 inhibition. This indicates that the regulatory effect of SNHG1 on EMT is dependent on the ULK1-Notch1 signaling pathway (Fig. 5h).

Thus, these findings establish SNHG1 as a critical modulator of EMT in Barrett’s epithelial cells through inhibition of autophagy and enhancement of the ULK1-Notch1 signaling interaction. The reversal of EMT phenotypes by RAPA and DAPT further confirms that the effects of SNHG1 are mediated via the ULK1-Notch1 axis, highlighting its potential as a therapeutic target in Barrett’s esophagus.

### SNHG1 modulates EMT in a Barrett’s esophagus mouse model (p16^flox/flox^/Kras^G12D^)

**Fig. 6:**
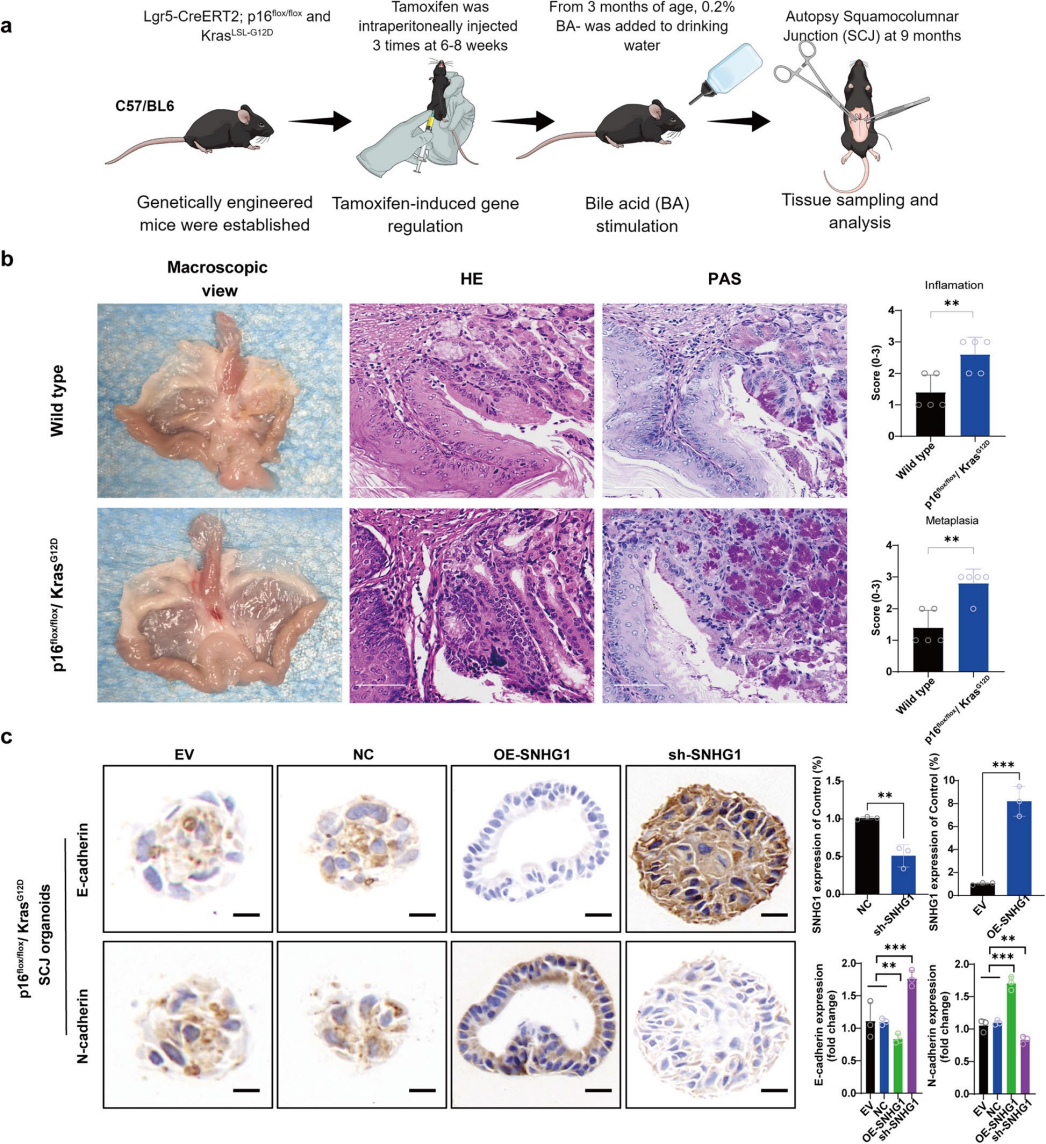
SNHG1 Modulates EMT in Squamocolumnar Junction (SCJ) Tissues of p16^flox/flox^/Kras^G12D^ Mice **a** Experimental design of the p16^flox/flox^/Kras^G12D^ genetically engineered mice. From 3 months of age, mice were treated with tamoxifen for gene regulation and exposed to 0.2% bile acid (BA) in drinking water until euthanasia at 9 months for tissue collection and analysis. **b** Histological analysis of inflammation and metaplasia in wild-type and p16^flox/flox^/Kras^G12D^ mice. Quantitative analysis revealed significantly higher inflammation and metaplasia scores in p16^flox/flox^/Kras^G12^^D^ mice compared to wild-type controls (*p* < 0.05 and *p* < 0.01). Scale Bar, 100 µm. **c** Immunohistochemical staining of EMT markers in SCJ organoids from p16^flox/flox^/Kras^G12D^ mice. Overexpression of SNHG1 (OE-SNHG1) resulted in reduced E-cadherin expression and increased N-cadherin levels, indicating enhanced EMT. Knockdown of SNHG1 (sh-SNHG1) reversed these effects, with increased E-cadherin and decreased N-cadherin expression. Scale Bar, 10 µm. The EV conditions refer to the empty vector, which serves as the overexpression control. NC refers to the normal control, which is shNotch1 control. We used one-way ANOVA with Tukey’s post hoc test for multiple group comparisons, and Student’s t-test or Mann-Whitney U test for two-group comparisons. The values are the means ± standard error of at least three separate studies

To validate our in vitro findings, we performed in vivo experiments using genetically engineered mice that spontaneously developed Barrett’s esophagus. Figure 6a illustrates the experimental design, in which p16^flox/flox^/Kras^G12D^ mice underwent tamoxifen-mediated gene activation starting at three months of age and were continuously exposed to 0.2% bile acid (BA) in their drinking water until euthanasia at nine months. Tissue samples were then collected for histological analysis.

Histological examinations showed that wild-type control mice did not display significant inflammation or metaplasia. In contrast, p16^flox/flox^/Kras^G12D^ mice exhibited notable tissue damage, characterized by pronounced inflammation and evident metaplastic changes. Quantitative scoring confirmed significantly elevated inflammation (*p* < 0.05) and metaplasia (*p* < 0.01) in the p16^flox/flox^/Kras^G12D^ group compared to wild-type controls (Fig. 6b).

To further investigate SNHG1’s regulatory role in EMT within Barrett’s metaplastic cells at the mouse SCJ, we generated organoids from mouse SCJ tissues and conducted targeted gene manipulation of SNHG1. Immunohistochemical analysis revealed that SNHG1 overexpression (OE-SNHG1) resulted in decreased E-cadherin and increased N-cadherin expression, indicative of EMT enhancement. Conversely, SNHG1 knockdown (sh-SNHG1) reversed these effects, significantly elevating E-cadherin levels while reducing N-cadherin expression (Fig. 6c).

These findings collectively underscore SNHG1’s critical role in modulating EMT processes and driving the progression of Barrett’s metaplasia within the p16^flox/flox^/Kras^G12D^ mouse model.

## Discussion

BE is a condition characterized by abnormal remodeling of esophageal mucosa, closely associated with chronic gastroesophageal reflux disease (GERD). Specialized intestinal-type Barrett’s epithelial cells commonly replace normal esophageal mucosa, significantly increasing the risk of esophageal adenocarcinoma [24]. Despite effective treatments such as radiofrequency ablation (RFA), recurrence remains frequent due to subsquamous intestinal metaplasia (SSIM), closely linked with EMT driven by chronic exposure to acidic bile salts [6, 25, 26]. Acidic bile salts influence several critical signaling pathways, including VEGF and Notch1, facilitating EMT and malignant transformation [13, 17]. Dysfunctional Notch signaling is widely recognized to accelerate cancer progression by influencing cell differentiation, proliferation, and invasiveness [27–29]. Our results specifically highlight the activation of Notch signaling as a crucial driver of EMT in Barrett’s epithelial cells exposed to acidic bile salts, reinforcing its significant role in BE progression toward adenocarcinoma.

Autophagy, an essential cellular degradation process, plays a central role in modulating EMT through interactions with the Notch1 signaling pathway [18, 30] Activation of autophagy facilitates the degradation of the Notch intracellular domain (NICD), a key transcriptional regulator of EMT and cancer metastasis [18]. Our study further elucidates this interaction by demonstrating that NICD directly interacts with and phosphorylates ULK1, thereby suppressing autophagy initiation. RAPA, an mTOR inhibitor known to activate autophagy, was shown to reduce NICD levels and suppress EMT markers effectively. Additionally, using DAPT, a γ-secretase inhibitor that inhibits Notch signaling, we validated the critical role of Notch signaling in modulating autophagy via ULK1 phosphorylation. Notably, the combined application of DAPT and RAPA significantly enhanced autophagy and effectively inhibited EMT compared to either treatment alone. Co-immunoprecipitation (Co-IP) assays confirmed the direct interaction between Notch1 and ULK1, underscoring the importance of the ULK1-Notch1 axis in regulating EMT and autophagy in BE cells.

Long non-coding RNAs (lncRNAs), defined as transcripts longer than 200 nucleotides, play essential roles in regulating gene expression across diverse biological contexts [31, 32]. Among them, SNHG1 has been previously implicated in tumor proliferation and metastasis, particularly in esophageal squamous cell carcinoma and gastric cancer, where it exerts oncogenic effects through pathways such as PI3 K/AKT or Wnt/β-catenin [27]. However, its role in BE has remained unexplored. Our study identifies SNHG1 as a novel driver of BE progression by promoting EMT through modulation of the ULK1-Notch1 axis. Functional experiments confirmed that altering SNHG1 expression significantly impacts EMT markers, cell migration, and autophagy status, both in vitro and in vivo.

In contrast to previous studies that independently examined autophagy and Notch1 signaling in BE or other cancers [33, 34], our findings mechanistically connect these two processes through ULK1, a key autophagy initiator. We demonstrate that ULK1 physically interacts with Notch1, and that this interaction is modulated by SNHG1, forming a unified regulatory circuit. This SNHG1–ULK1–Notch1 axis coordinates the suppression of autophagy and activation of EMT, a mechanism not previously reported in BE. Thus, our work provides a new dimension to the functional repertoire of SNHG1 and adds mechanistic depth to our understanding of EMT regulation under chronic acidic bile salt exposure.

The implications of these findings are twofold. First, they suggest that SNHG1 could serve as a predictive biomarker for BE progression, particularly in identifying patients at risk for dysplasia following RFA treatment. Second, the observed reversibility of SNHG1-induced EMT via RAPA or DAPT highlights potential therapeutic strategies for halting or reversing BE evolution. Targeting SNHG1 directly, or its downstream signaling components ULK1 and Notch1, could offer new avenues for chemopreventive intervention and improve the long-term management of BE. These insights support future development of lncRNA-based diagnostics and therapeutics tailored to the molecular subtype of Barrett’s epithelium.

This study has several limitations. First, our findings rely heavily on the BAR-T and CP-C cell lines, immortalized Barrett’s epithelial models, which may not fully capture the complexity and heterogeneity of human BE. Using additional models, such as patient-derived organoids, could enhance translational relevance. Second, although the p16^flox/flox^/Kras^G12D^ mouse model provides valuable insights, it cannot entirely replicate the multifactorial and heterogeneous nature of BE in humans, particularly regarding environmental exposures and genetic diversity. Third, clinical validation of the SNHG1-ULK1-Notch1 axis in patient samples is currently lacking, limiting direct clinical applicability. Further studies utilizing human tissues are necessary to confirm clinical relevance. Methodologically, Co-IP assays, while valuable, may not definitively capture transient or direct protein interactions, and additional validation methods are needed to confirm specificity.

Collectively, our findings uncover novel molecular pathways underlying BE pathogenesis, emphasizing the significance of the SNHG1-ULK1-Notch1 axis as a promising therapeutic target. This research provides foundational insights for developing personalized therapeutic strategies aimed at preventing recurrence and progression in patients with Barrett’s esophagus.

## Materials and methods

### Ethical approval of clinical studies and animal studies

The study was approved by the Ethics Committee of West China Hospital, Sichuan University, and all participants signed informed consent forms (2020(1094)). All animal experimental protocols were approved by the Ethics Committee of West China Hospital, Sichuan University (Approval number: No.20220107005). All animal experiments were in accordance with the guide for the care and use of laboratory animals established by the United States National Institutes of Health.

### Clinical specimen collection and classification

Endoscopic biopsy specimens were obtained from patients with histologically confirmed BE at West China Hospital, Sichuan University. Written informed consent was obtained from all participants prior to tissue collection. For each patient, biopsy samples were obtained from the squamocolumnar junction during routine surveillance endoscopy and stored in RNAlater (Thermo Fisher Scientific, USA) at −80 °C until RNA extraction. Based on longitudinal histopathologic evaluation and clinical follow-up over a minimum of 24 months, patients were categorized into three groups: (1) indolent/non-progressors, (2) progressed to low-grade dysplasia (LGD), and (3) progressed to high-grade dysplasia (HGD). Only initial diagnostic biopsy specimens were used for molecular analysis. SNHG1 expression was quantified via quantitative real-time PCR (qPCR), and its association with progression status was statistically evaluated.

### Cell culture and treatment

Barrett’s epithelial cells (BAR-T and CPC cells) were purchased from ATCC (Virginia, USA). These cells were grown in 1640 medium from Hyclone (Logan, UT, USA), supplemented with 10% fetal bovine serum, and were maintained in an incubator at 37 °C in an atmosphere containing 5% CO_2_.

### Cell transfection

The SNHG1 overexpression plasmids and siRNA were synthesized by Genechem (shanghai, China). Barrett’s epithelial cells cultured in DMEM were transfected with Lipofectamine 3000 (Invitrogen, Carlsbad, CA, USA) once they reached 70%–80% confluence, and the transfection was allowed to proceed for 36 hours.

### Scratch wound assay

Initially, 6-well plates were used to seed Barrett’s epithelial cells, which were then incubated for 24 hours. Next, using a sterile 200 μL pipette tip, each well’s cells were gently scraped. After three PBS rinses, cells were cultured for a further twenty-four hours in DMEM with different treatments according to their groups. Two times were recorded for the images: the first time at 0 hours and the second time 24 hours later. Images were captured using a inverted microscope (Leica DMi8, Germany). Using ImageJ software, the wound area was defined and measured [35].

### Trans-well assay

A Millipore trans-well chamber (Burlington, MA, USA) was used to hold a cell suspension at a concentration of 1 × 10^5 cells/mL (200 μL) in the upper compartment, and 800 μL of specialized media were placed in the lower compartment for every experimental group. After a 24-hour incubation period, the upper chamber cells were extracted, and the chambers were fixed with 4% paraformaldehyde for 30 minutes. Following that, staining was done for 20 minutes using a 0.1% crystal violet solution. Finally, the stained cells in five randomly selected sections of each well were quantified using an inverted microscope (Leica DMi8, Germany) [36].

### Immunofluorescence staining

BAR-T and CPC cells were seeded onto sterilized glass coverslips in 24-well plates and allowed to adhere overnight. Following treatment, cells were washed twice with PBS and fixed with 4% paraformaldehyde for 15 minutes at room temperature. Cells were then permeabilized using 0.1% Triton X-100 for 10 minutes and blocked with 5% bovine serum albumin (BSA) for 1 hour. Subsequently, cells were incubated with primary antibodies overnight at 4 °C. After washing with PBS, cells were incubated with secondary antibodies for 1 hour at room temperature in the dark. Nuclei were counterstained with DAPI (1 μg/mL) for 5 minutes. Coverslips were mounted with anti-fade mounting medium and observed using a fluorescence microscope (Olympus IX71, Japan). Fluorescence intensity was quantified using ImageJ software.

### Western blotting

RIPA buffer (Beyotime, Shanghai, China) was utilized to lyse the Barrett’s epithelial cells. To quantify the protein levels, we employed the BCA protein assay kit (Beyotime, Shanghai, China). The separation of target proteins by their molecular weights was achieved through sodium dodecyl sulfate-polyacrylamide gel electrophoresis, followed by their transfer onto PVDF membranes (Millipore, Burlington, MA, USA). After that, a 2-hour room-temperature blocking stage was completed. After applying primary antibodies, secondary antibodies coupled with horseradish peroxidase were added to identify proteins. Enhanced chemiluminescence was used as the HRP substrate (4A Biotech, Beijing, China) to see the proteins. We employed ImageJ software (National Institutes of Health, USA) to do quantitative protein analysis [37].

### Antibodies

The following antibodies were used: E-cadherin (20874-1-AP, proteintech, Wuhan, China), N-cadherin (A19083, ABclonal, Wuhan, China), MMP9 (10375-2-AP, proteintech, Wuhan, China), MMP2 (66366-1-Ig, proteintech, Wuhan, China), Vimentin (A19607, ABclonal, Wuhan, China), Snail (3879S, Cell Signaling Technology, Danvers, MA, USA), Jagged1 (70109S, Cell Signaling Technology, Danvers, MA, USA), Notch1(3608S, Cell Signaling Technology, Danvers, MA, USA; ab52627, Abcam, Cambridge, UK), p62 (CST88588S, Cell Signaling Technology, Danvers, MA, USA), LC3 (ab192890, Abcam, Cambridge, UK), p-ULK1 (14202S, Cell Signaling Technology, Danvers, MA, USA), ULK1 (8054S, Cell Signaling Technology, Danvers, MA, USA; ab167139, Abcam, Cambridge, UK), β-actin (66009-1-Ig, proteintech, Wuhan, China).

### Quantitative Real-Time PCR

Total RNA was extracted from Barrett’s epithelial cells using TRIzol reagent (Invitrogen, USA) according to the manufacturer’s instructions. RNA concentration and purity were measured using a NanoDrop 2000 spectrophotometer (Thermo Fisher Scientific, USA). Complementary DNA (cDNA) was synthesized using the PrimeScript™ RT Reagent Kit with gDNA Eraser (Takara, Japan). Quantitative real-time PCR was performed using TB Green™ Premix Ex Taq™ II (Takara, Japan) on a CFX96 Real-Time PCR Detection System (Bio-Rad, USA). Relative gene expression was calculated using the 2^-ΔΔCt method, with β-actin as the internal control. Each reaction was conducted in triplicate. The primer sequences were listed in supplementary materials.

### Transmission Electron Microscopy (TEM)

To visualize autophagosomes, BAR-T cells were fixed in 2.5% glutaraldehyde (Sigma-Aldrich, USA) in 0.1 M phosphate buffer (pH 7.4) at 4 °C overnight. Samples were post-fixed with 1% osmium tetroxide for 2 hours, dehydrated through a graded ethanol series, and embedded in epoxy resin. Ultrathin sections (70 nm) were obtained using an ultramicrotome (Leica EM UC7, Germany) and mounted on copper grids. Sections were stained with uranyl acetate and lead citrate, then examined under a transmission electron microscope (Hitachi HT7700, Japan). Autophagic vacuoles were identified and quantified in randomly selected fields at 20,000× magnification.

### LC3 autophagic flux detection via dual fluorescent reporter

To assess autophagic flux, BAR-T cells were transfected with an mRFP-GFP-LC3 tandem fluorescent-tagged plasmid (Genechem, Shanghai, China) using Lipofectamine 3000 (Invitrogen, USA) according to the manufacturer’s instructions. After 24 hours of transfection, cells were treated under indicated conditions (e.g., acidic bile salts). The mRFP-GFP-LC3 reporter enables discrimination between autophagosomes (GFP+/RFP+, yellow puncta) and autolysosomes (GFP−/RFP+, red puncta), as the GFP signal is quenched in the acidic lysosomal environment. Cells were imaged using a confocal laser scanning microscope (Leica TCS SP8, Germany). Quantification of LC3 puncta was performed using ImageJ.

### Protein-protein docking analysis

To predict the potential physical interaction between ULK1 and Notch1, protein–protein docking analysis was performed using Discovery Studio 4.0 software (BIOVIA, San Diego, CA, USA). The three-dimensional structure of ULK1 was obtained from the Protein Data Bank (PDB ID: 4WNO), which represents the crystal structure of the ULK1 kinase domain in complex with a small-molecule inhibitor. As a full-length experimentally resolved structure of Notch1 is currently unavailable, a high-confidence predicted model from the AlphaFold database (ID：AF-P46531-F1) was employed for docking analysis. Rigid-body docking was conducted using the ZDOCK module, which evaluates candidate complexes based on shape complementarity, electrostatic interactions, and desolvation energy. The top-scoring complex was visualized in three dimensions, and the predicted binding interface was analyzed for hydrogen bond formation and spatial compatibility to infer potential interaction sites and binding modes between ULK1 and Notch1.

### Co-IP assay

Barrett’s epithelial cells were cultured for 1 hour in IP lysis buffer (Beyotime, Shanghai, China). The supernatant was produced by centrifuging cell lysates at 12,000 RPM for 10 minutes to separate them. A 10 μL portion of the supernatant was set aside for measuring the protein content, while the rest was gently mixed with specific antibodies or normal IgG from the same species and incubated at 4 °C for the entire night. The samples were incubated for 4 hours with the addition of Protein A/G magnetic beads (Thermo Fisher Scientific, Waltham, USA) and a wash with IP lysis solution. Subsequently, the beads were washed three times with PBS and twice with IP lysis solution. 2× SDS loading buffer (Beyotime, Shanghai, China), which was used to elute and denature the precipitated proteins for ten minutes at 100 °C. Ultimately, Western blotting was used to examine the proteins [38, 39].

### WGCNA analysis

To better understand the pathogenesis of Barrett’s esophagus patients, we downloaded the GSE26886 dataset from the GEO database (https://www.ncbi.nlm.nih.gov/geo/). In the downstream analysis, we included the expression data of Barrett’s esophagus patients (*n*=20) and Control samples (biopsies from patients with normal esophageal squamous epithelium, *n*=19). R package WGCNA (version 1.70–3) was used to explore the eigengenes closely related to the occurrence and development of Barrett’s esophagus and the critical interaction between eigengenes. Firstly, we use the pickSoftThreshold function to filter the optimal soft threshold for weighted correlation algorithm calculations from 1–20. Subsequently, using the optimal soft threshold of 4 as the power value, the correlation coefficients between genes were multiplied to obtain the adjacency matrix of all genes. The Tree method with deepSplit was used to identify and divide each gene module, and the minimum height of the merge module was set to 0.25, while the minimum module size for module detection was set to 30. The threshold for building the network is set to the default value of 0.02, and the resulting interactions are considered to have regulatory relationships. Cytoscape software (version 3.9.1) was used to construct the core regulatory network of Barrett’s esophagus patients.

### Animal model and treatment protocol

Genetically engineered Lgr5-CreERT2, IL1B^tg^; p16^flox/flox^; Kras^LSL-G12D^; mice (C57BL/6 background) were obtained from GemPharmatech Co., Ltd. (Nanjing, China) and bred in a pathogen-free animal facility at West China Hospital. Mice were genotyped via PCR prior to study initiation. At 3 months of age, gene recombination was induced via intraperitoneal injection of tamoxifen (75 mg/kg/day, Sigma-Aldrich) for five consecutive days to activate Kras^G12D^ expression and knock out p16 expression.

Following recombination, mice were supplemented with 0.2% deoxycholic acid (DCA, Sigma-Aldrich) in drinking water, for six months to induce chronic bile reflux conditions. Mice were monitored weekly for weight, behavior, and general health. At the end of the exposure period (9 months of age), mice were euthanized, and the SCJ tissues were harvested for H&E staining, immunohistochemistry, and organoid culture. Histological scoring of inflammation and metaplasia was performed by two blinded gastrointestinal pathologists according to established criteria.

### Mouse SCJ-derived organoid culture and SNHG1 manipulation

Organoids were established from SCJ tissues as previously described with modifications. Briefly, harvested tissues were washed in cold PBS and incubated with 10 mM EDTA for 30 minutes at 4 °C to release epithelial crypts. After mechanical dissociation and filtration, crypts were embedded in Matrigel (Corning, 356231) and cultured in Advanced DMEM/F12 medium supplemented with: B27 supplement (1×, Gibco), N2 supplement (1×, Gibco), 50 ng/mL EGF (PeproTech), 100 ng/mL Noggin (PeproTech), 100 ng/mL R-spondin1 (PeproTech), 10 mM Nicotinamide, 1.25 mM N-acetylcysteine, 10 μM Y-27632 (for initial plating only). Lentiviral constructs for SNHG1 overexpression or knockdown (shRNA) were packaged using 293 T cells and transduced into organoids at passage 2. Organoids were harvested for immunohistochemical analysis of EMT markers (E-cadherin, N-cadherin), and images were quantified using ImageJ software.

### Statistical analysis

For the statistical analysis in this study, SPSS 24.0 and GraphPad Prism 7.0 were used. Comparisons were performed using the Kruskal-Wallis H test with Bonferroni’s adjustment for ranked data. The measurement data were presented as means ± standard deviations, and Tukey’s post hoc test was used after one-way analysis of variance (ANOVA) for multiple group comparisons. *P* < 0.05 was deemed as the statistically significant threshold. we define significance levels as follows: *** *p* < 0.001, ** *p* < 0.01, and * *p* < 0.05.

## Supplementary Information

Supplementary Material 1.

## Data Availability

The corresponding author will provide the original data used to support the findings of this study upon reasonable request.

## Abbreviations

AB30M: Acidic bile salts for 30 minutes
AB14D: Acidic bile salt medium for 30 minutes a day for 14 days
Co-IP: Co-immunoprecipitation
EMT: Epithelial-mesenchymal transition
ESCC: Esophageal squamous cell carcinoma
ZEB1/2: E-box-binding homeobox 1/2
lncRNAs: Long non-coding RNAs
MMP: Matrix metalloproteinase
mTOR: Mammalian target of ra-pamycin
NICD: Notch1 intracellular domain
RFA: Radiofrequency ablation
RAPA: Rapamycin
SNHG1: Small nucleolar RNA host gene 1
SSIM: Sub-squamous intestinal metaplasia
ULK1: UNC-52-like kinase 1
VEGF: Vascu-lar-endothelial-growth-factor
WGCNA: Weighted correlation network analysis
BE: Barrett’s esophagus
GERD: Gastroesophageal reflux disease
EAC: Esophageal adenocarcinoma
CDH1: Cadherin 1
qPCR: Quantitative Polymerase Chain Reaction
TEM: Transmission Electron Microscopy
SCJ: Squamocolumnar Junction
ESE: Esophageal squamous epithelium
