# Supplementary material for: LncRNA small nucleolar RNA host gene 1 (SNHG1) mediates acidic bile salt-induced EMT via the ULK1-Notch1 axis in Barrett’s esophagus
Source: Mol Biomed. 2025 Jul 9;6:49. doi: 10.1186/s43556-025-00285-4 (PMC12238433; doi:10.1186/s43556-025-00285-4)
Supplement: Supplementary file 1 — Supplementary Material 1. [file 43556_2025_285_MOESM1_ESM.pdf]

LncRNA small nucleolar RNA host gene 1 (SNHG1) mediates acidic bile salt-induced EMT via the ULK1-Notch1 axis in Barrett's esophagus

Jianfeng Zhou[1, #], Rongyan Zhao[1, #], Zixiang Li[1, 2, #], Xuelan Ma[1, 2], Wenke Jin[1, 2], Yong Yuan[1], Ning Li[3, *], Bo Liu[1, *]Yushang Yang[1, *]

[1]Department of Thoracic Surgery, Department of Biotherapy, Cancer Center and State Key Laboratory of Biotherapy, West China Hospital, Sichuan University, Chengdu 610041, China.

[2]Sichuan Engineering Research Center for Biomimetic Synthesis of Natural Drugs, School of Life Science and Engineering, Southwest Jiaotong University, Chengdu 610031, China

[3]School of Traditional Chinese Materia Medica, Key Laboratory of Innovative Traditional Chinese Medicine for Major Chronic Diseases of Liaoning Province, Key Laboratory for TCM Material Basis Study and Innovative Drug Development of Shenyang City, Shenyang Pharmaceutical University, Shenyang 110016, China

[#]These authors made equal contributions to this work.

*Corresponding authors, E-mail addresses: dryangysh@scu.edu.cn (Yushang Yang); liubo2400@163.com (Bo Liu); liningsypharm@163.com (Ning Li).

Supplement

Supplementary Fig. 1. SNHG1 Potentially Mediates the Interaction between ULK1 and Notch1 in Barrett's Esophageal Cells.

Supplementary Fig. 2: Off-target Effect Reversal Experiment of siRNA

Supplemental material: Sequence Information

Supplemental material: Prognostic Role of Notch1 in Esophageal Adenocarcinoma

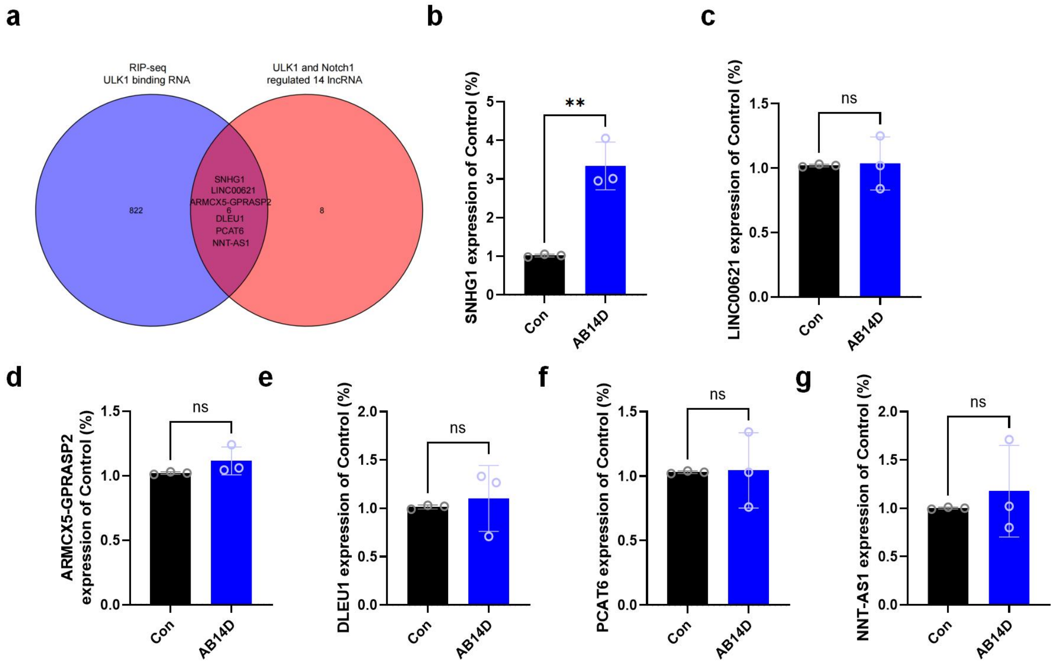

Supplementary Fig. 1. SNHG1 Potentially Mediates the Interaction between ULK1 and Notch1 in Barrett's Esophageal Cells

(a) Venn diagram showing the intersection of ULK1-binding lncRNAs identified via RNA immunoprecipitation sequencing (RIP-seq, blue circle, n = 822) and Notch1/ULK1 co-regulated lncRNAs (red circle), revealing seven candidate lncRNAs: SNHG1, LINC00621, ARMCX5-GPRASP2, DLEU1, PCAT6, NNT-AS1, and one unnamed transcript.

(b–g) Quantitative RT-PCR validation of the expression levels of the seven candidate lncRNAs in Barrett's esophageal cells treated with acidic bile salts (AB14D) versus control. Among the candidates, SNHG1 showed a significant increase in expression upon AB14D exposure ($P < 0.01$, unpaired t-test), whereas the expression of LINC00621 (c), ARMCX5-GPRASP2 (d), DLEU1 (e), PCAT6 (f), and NNT-AS1 (g) did not show significant changes (ns, not significant).

Data are presented as mean ± SEM (n = 3). Statistical comparisons were performed using unpaired two-tailed Student's t-tests.

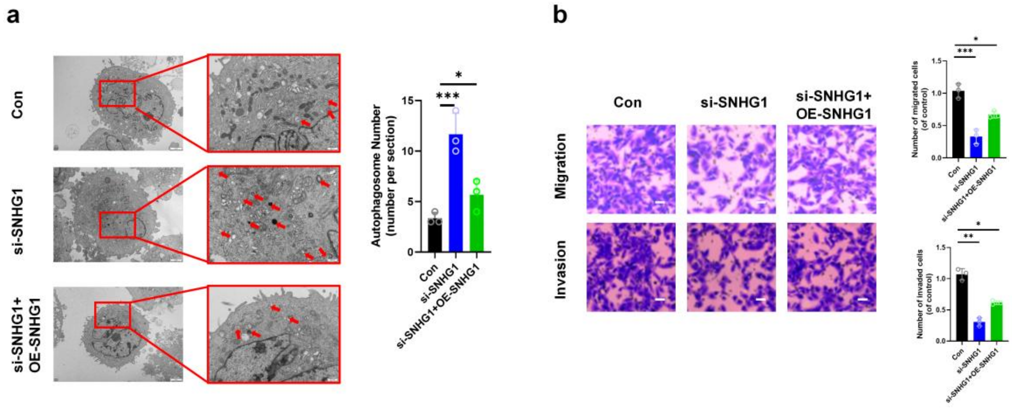

Supplementary Fig. 2. Off-target Effect Reversal Experiment of siRNA

(a) Transmission electron microscopy (TEM) was used to visualize autophagosomes in BAR-T cells transfected with siRNA targeting SNHG1 (si-SNHG1), an siRNA-resistant SNHG1 overexpression plasmid (OE-SNHG1), or both (si-SNHG1 + OE-SNHG1). Compared to control cells (Con), SNHG1 knockdown significantly increased the number of autophagic vesicles (red arrowheads), indicating enhanced autophagy. Co-transfection with OE-SNHG1 partially reversed this phenotype, as shown by a reduced number of autophagosomes. Quantification of autophagosome number per cell section is shown on the right. Data are presented as mean ± SEM (n = 3); ***P < 0.001, one-way ANOVA with Tukey's post hoc test.

(b) Transwell migration and invasion assays were performed to evaluate the phenotypic effects of SNHG1 silencing and rescue. SNHG1 knockdown markedly impaired both migratory and invasive capacity of BAR-T cells. However, co-expression of SNHG1 (siRNA-resistant) significantly restored cell motility. Representative images are shown on the left, and quantification of migrated and invaded cells per field is shown on the right. Scale Bar, 100 μm. Data are presented as mean ± SEM (n = 3); *P < 0.05, **P < 0.01, one-way ANOVA with Tukey's post hoc test.

Supplemental material: Sequence Information

1. qPCR primers

Gene name	Forward Primer	Reverse Primer
SNHG1	GCCAGCACCTTCTCTCTAAAGC	GTCCTCCAAGACAGATTCCATTTT
β-actin	CATGTACGTTGCTATCCAGGC	CTCCTTAATGTCACGCACGAT

2. siRNAs

siRNAs	sense sequence	anti-sense sequence
si-Notch1#1	5'-CUUUGUUUCAGGUUCAGUAtt-3'	5'-UACUGAACCUGAAACAAAGtt-3'
si-Notch1#2	5'-CAAAGACAUGACCAGUGGCtt-3'	5'-GCCACUGGUCAUGUCUUUGtt-3'
si-SNHG1#1	5'-CCAGCAUCUCAUAAUCUAUtt-3'	5'-AUAGAUUAUGAGAUGCUGGaa-3'
si-SNHG1#2	5'-CCUUCUCUCUAAAGCCCAAtt-3'	5'-UUGGGCUUUAGAGAGAAGGtg-3'

3. SNHG1 Overexpressed plasmid

Gene names and species	SNHG1-human
GeneID:	23642
transcript	NR_003098;1137bp<br>NR_152582;1047bp<br>NR_152583;867bp<br>NR_152577;1074bp<br>NR_152585;1484bp<br>NR_152575;1535bp<br>NR_152580;1039bp<br>NR_152578;1043bp<br>NR_152576;1527bp<br>NR_152581;918bp<br>NR_152579;1071bp<br>NR_152584;1316bp

Supplemental material: Prognostic Role of Notch1 in Esophageal Adenocarcinoma

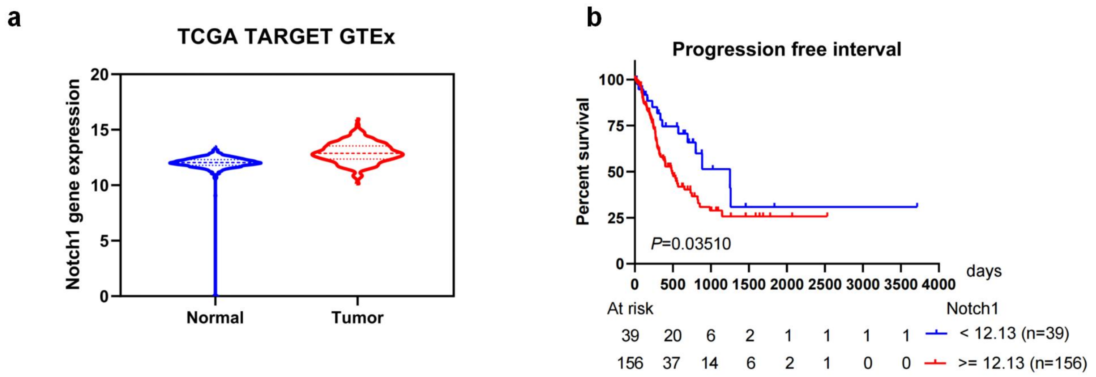

(a) Violin plot comparing Notch1 gene expression levels between normal esophageal tissues (blue) and esophageal adenocarcinoma (EAC) tumors (red) using data from the TCGA, TARGET, and GTEx datasets. Notch1 expression was significantly higher in tumor tissues, indicating a potential role in EAC pathogenesis.

(b) Kaplan–Meier analysis of progression-free interval (PFI) in EAC patients stratified by Notch1 expression levels (cutoff = 12.13). Patients with high Notch1 expression (≥12.13, red curve) exhibited significantly shorter PFI compared to those with low expression (<12.13, blue curve), suggesting that elevated Notch1 is associated with worse clinical outcomes. P = 0.03510, log-rank test. The number of patients at risk is indicated below the curve.
